# Strategies for an effective tobacco harm reduction policy in Indonesia

**DOI:** 10.4178/epih/e2014035

**Published:** 2014-12-10

**Authors:** Fariz Nurwidya, Fumiyuki Takahashi, Hario Baskoro, Moulid Hidayat, Faisal Yunus, Kazuhisa Takahashi

**Affiliations:** 1 Department of Respiratory Medicine, Juntendo University Graduate School of Medicine, Tokyo, Japan; 2 Department of Pulmonology and Respiratory Medicine, University of Indonesia Faculty of Medicine, Jakarta, Indonesia

**Keywords:** Policy, Tobacco, Indonesia

## Abstract

Tobacco consumption is a major causative agent for various deadly diseases such as coronary artery disease and cancer. It is the largest avoidable health risk in the world, causing more problems than alcohol, drug use, high blood pressure, excess body weight or high cholesterol. As countries like Indonesia prepare to develop national policy guidelines for tobacco harm reduction, the scientific community can help by providing continuous ideas and a forum for sharing and distributing information, drafting guidelines, reviewing best practices, raising funds, and establishing partnerships. We propose several strategies for reducing tobacco consumption, including advertisement interference, cigarette pricing policy, adolescent smoking prevention policy, support for smoking cessation therapy, special informed consent for smokers, smoking prohibition in public spaces, career incentives, economic incentives, and advertisement incentives. We hope that these strategies would assist people to avoid starting smoking or in smoking cessation.

## INTRODUCTION

There is increasing evidence and data regarding morbidity and mortality caused by smoking in various countries. In fact, every ton of tobacco consumed results in one death [1]. It has been reported that more than 62% of adult males in Indonesia regularly smoke, and this is implicated in an increase in the prevalence of non-communicable diseases and a high demand on health services [2]. The prevalence of smoking in young adult Indonesian males increased from 53.4% in 1995 to 63.1% in 2004, while the proportion of Indonesian females who smoke rose from 1.7% in 1995 to 4.5% in 2004 [3]. In addition, the total number of cigarettes consumed increased from 33 billion in 1970 to 217 billion in 2004 [3]. According to the Global Adult Tobacco Survey in Indonesia in 2011, the prevalence of smoking was 67.0% in males and 2-7% in females [4]. After the Indonesian government rejected a proposal to increase excises on cigarettes, domestic cigarette production in Indonesia was predicted to increase in 2014 to between 355 and 360 billion cigarettes [5]. Indonesia’s cigarette export in 2010 was 55,150,912 kg, worth USD 428,001,559, while cigarette import was 254,164 kg, worth USD 4,880,791 [6].

Anti-smoking campaigns, such as on television, have tried to discourage people from smoking, by disseminating information about the short- and long-term health implications of smoking [7]. Here, we propose various policy options for future tobacco control. We also aligned our proposed strategy with the World Health Organization (WHO) MPOWER strategy as part of measures to implement the WHO Framework Convention on Tobacco Control. Briefly, WHO MPOWER strategies are: monitor tobacco use and prevention policies; protect people from tobacco smoke; offer help to quit tobacco use; warn about the dangers of tobacco; enforce bans on tobacco advertising, promotion and sponsorship; and raise taxes on tobacco [8]. Strategies that were developed in this proposal were based on research of literature in the PubMed database, as well as on experiences from various countries that have already implemented parts of a tobacco harm reduction strategy.

## PROPOSED POLICIES FOR TOBACCO ADVERTISEMENT INTERFERENCE

Massive commercial advertisement of tobacco has resulted in an increasing number of smoking citizens in Indonesia, particularly in the adolescent subpopulation, who are still in pursuit of their self-identity and who are being identified by Indonesian tobacco companies as an important market to maintain company profits [9]. Therefore it is crucial to start tobacco control with advertisement interference. The first effective strategy is the inclusion of images of cancer and birth defects on cigarette packs. Furthermore, a policy for health warnings to be displayed in cigarette packets has been executed by the Singapore government and gave satisfactory results in reducing the number of smokers in the country [10]. However, the inclusion of details of a tobacco telephone advice line on cigarette packs is not sufficient to provide clear information about the risks of tobacco consumption. In contrast, it has recently been reported that the use of pictorial warning labels on cigarette packaging could result in implicit emotional directives evoked by such warnings that lead to the intention to quit smoking [11,12].

Cigarette advertising should only depict patients who are under chemotherapy for cancer due to smoking. Patients who have undergone radiotherapy or surgery can also be considered. Tobacco companies are unlikely to employ such realistic advertising. If necessary, only models who have smoking-related illnesses may be allowed to participate in cigarette advertising. Therefore, the advertisement of cigarettes should, as much as possible, avoid the element of ‘entertainment’.

The first two strategies above can be categorized as ‘show as it is’ policy. These proposals match the WHO MPOWER strategy points of ‘enforce bans on tobacco advertising’ and ‘warn about the dangers of tobacco’.

## CIGARETTE PRICING POLICY

Regulation of cigarette price is an important strategy to reduce tobacco consumption. It has been reported that increasing cigarette prices by around 10% would result in a reduction of demand for cigarettes of around 3-5% in developed countries and around 6-10% among developing and under-developed countries [13]. It is common to regulate cigarette prices by increasing cigarette tax. Recent evidence also suggests that to maximize the effectiveness of tobacco taxation policies, countries should apply specific excise tax structures to reduce variation in tobacco price, which would prevent smokers from using an alternative, less-expensive brand [14]. This step is in alignment with the WHO MPOWER ‘raise taxes on tobacco’ strategy.

## ADOLESCENT SMOKING PREVENTION POLICY

The young adult population is at risk for the initiation of and progression to established tobacco use [15]. The initiation of cigarette smoking usually occurs between late childhood and young adolescence [16]. A policy to deter adolescents from smoking is urgently needed, because smoking during adolescence is implicated in long-term health outcomes. Multiple strategies should be considered and combined, such as reducing the overall density of tobacco outlets [17], enforcement of smoking rules in family cars [18], home smoking restriction policy [19], and school regulations to ban smoking [20]. This action forms part of the WHO MPOWER ‘protect people from tobacco smoke’ strategy. However, a remaining problem that should be addressed is how to increase and maintain public self-awareness for adolescents to be able to maintain a non-smoking status.

## SPECIAL INFORMED CONSENT FOR SMOKERS: INDIVIDUAL RESPONSIBILITY

Citizens who wish to purchase cigarettes must first sign a so-called informed consent. Informed consent generally refers to a statement to be signed by patients before medical treatment or surgery. Regarding cigarette restriction policy, informed consent would apply to citizens who want to smoke, with statements as follows: (a) ‘I understand all the consequences of smoking, such as heart disease, cancer, and fetal abnormalities’; (b) ‘I have secured the availability of food, milk, clothing, and education for my family, and these will not be affected by the purchase of cigarettes’; (c) ‘I am prepared to deal with any illness and loss of material and immaterial wellbeing due to cigarette smoking’; (d) ‘I will not sue anyone because of disease that may occur as a result of my smoking’; and (e) ‘I will not use health care insurance benefits provided by the government’. The application of informed consent in this regard may be termed as ‘responsibility’. This strategy represents the implementation of the WHO MPOWER ‘monitor tobacco use and prevention policies’ component. The potential limitation of this strategy will be the time that will be required for millions of smokers in Indonesia to give this informed consent.

## SUPPORT FOR SMOKING CESSATION THERAPY

Smokers need a supportive environment when they decide to quit smoking. Support for those who want to stop smoking could be given in various ways, such as via a smoking cessation campaign, an ex-smoker community gathering, and, most importantly, the integration of smoking cessation treatment into national health insurance schemes. In Indonesia, drugs such as varenicline are still not covered by national health insurance, and there are only a limited number of specialized smoking cessation clinics. Currently, a smoking cessation clinic is located at the Persahabatan General Hospital, a University of Indonesia-affiliated national referral hospital for respiratory diseases at Jakarta. The government should consider incorporating smoking cessation medicine into the national health insurance system, as well as increasing the number of smoking cessation clinics. This program will represent the implementation of the ‘offer help to quit tobacco use’ WHO MPOWER strategy. Potential barriers to this approach will be choosing the most cost-effective smoking cessation regimens to be integrated into insurance coverage. Moreover, the possible adverse effects of the chosen drug(s) should be clarified.

One good example comes from Korea, which launched the Quitline Service in 2006, consisting of programs including private counseling and support for people to quit smoking, collaboration with hundreds of smoking cessation clinics across the nation, and offering a free consultation service as well as free smoking cessation-related medicine to people who intend to quit smoking [21].

## SMOKING PROHIBITION IN PUBLIC SPACES

Since 2005, Jakarta has officially implemented a local anti-smoking act; however, the main problem with this is law enforcement in the field. Societal awareness of a smoke-free environment is still lacking. Recently, a community-based anti-tobacco movement named the Coalition for a Smoke-free Jakarta expressed their support for the Provincial Government of Jakarta by joining officers in the field to help monitor and prevent people smoking in public spaces such as public buildings, streets, and restaurants, using persuasive approaches.

Another issue is that the anti-smoking laws apply only in certain provinces of Indonesia. Anti-smoking legislation at the national level is urgently required. This would be expected to increase smokers’ motivation to quit, based on previous research in Turkey that assessed changes in smoking behaviors after the introduction of anti-smoking legislation [22] and found that the new law encouraged smokers to stop smoking.

### Economic incentives

Cigarette sellers should receive counseling about the possibility of working in alternative trading businesses. The government should consider persuading banks to ease lending procedures for tobacco merchants who want to switch to other commodities. Local governments usually already know the number of tobacco farmers in the area. One possible program is, for example, for the local agricultural officer to routinely visit tobacco farmers in order to encourage and counsel them so they can understand other farm commodities. Examples of former tobacco farmers that have successfully switched to other agricultural commodities will increase the awareness and the motivation of other tobacco farmers to leave tobacco farming. In Zimbabwe, for example, a case study found that large commercial farmers were actively using tobacco revenue to develop new enterprises specifically to lessen their dependence on tobacco [23]. Moreover, many tobacco farmers are poor, and now is the time for them to increase their welfare by planting alternative agricultural commodities enriched with high protein and high nutrition.

Tobacco farming has many negative consequences for the health and well-being of farmers and other tobacco workers [24]. Therefore, it is necessary to approach several industries, for example, the coffee, cocoa, tea, palm oil, food and even herbs industries, to encourage and prepare them to provide work opportunities for cigarette factory workers who want to work in a non-tobacco factory. The potential handicap for this strategy is the national budget that will be required to open a new sector of industry and allow for new job vacancies.

The tobacco industry often argues that closing cigarette factories will lead to the loss of working opportunities for thousands of workers. However, this argument is manipulative and easily rebutted. For example, the food industry regularly indicates its readiness to recruit cigarette factory workers. This step is best carried out five years prior to the closure of a cigarette factory, since gradual phasing in of changes is important. This is the key to successful policies that we classify as ‘economic incentives’. These steps are also in alignment with the WHO MPOWER strategy of ‘monitor tobacco use and prevention policies’.

### Career incentives

Job recruitment strategies also play an important role in tobacco harm reduction, such as the addition of the criterion of ‘no smoking’ in job advertisements. This criterion could be a morale booster for job seekers who are willing to quit smoking. Furthermore, in the promotion of employees, the criterion of ‘no smoking’ can be used in assessment processes. The company management is expected to give a small reward to staff members who do not smoke. This will establish a new mind-set: that not smoking is a positive achievement. We classify this step as ‘career incentives’. This proposal also matches the ‘monitor tobacco use and prevention policies’ point in the WHO MPOWER.

### Advertisement incentive

The next incentive is the allocation of tobacco tax to the funding of anti-smoking advertisements. This is important, because tobacco advertising is backed by large capital. Increased sales of cigarettes should be accompanied by an increase in anti-smoking public service announcements, in order to apply a ‘brake’ to the rate of cigarette consumption. We categorize this as an ‘advertisement incentive’. Nevertheless, some limitations should be anticipated, in the form of major tobacco company interference with governmental officials, which may result in political resistance to this strategy.

The concept that smokers are ‘unique citizens’ may previously have been regarded as unconventional. We consider smokers as ‘unique citizens’, because despite being in a healthy condition, smokers often consciously take on the multiple risks of smoking, for themselves and those closely surrounding them, but then regret smoking when the risk becomes a reality. It is very rare to meet people with lung cancer who say, “I do not regret having a smoke”: in other words, nearly all patients who have previously smoked would eventually regret smoking. Because of the uniqueness of this scenario, therefore, we must begin to introduce the concept of ‘unique citizens’. These ‘unique citizens’ may also be considered to have ‘unique rights’, such as the reduction of employment opportunities and access to government health insurance for the poor. We hope not to limit the scope of our ideas regarding the possibilities of tobacco elimination and harm reduction strategies, which include the prohibition of the sale of tobacco products through on-line methods [25]. We envision an ‘Indonesia Free from Smoking’.

## CONCLUSION

The possibilities that we have introduced must be considered, discussed, and then given broad application in the field, with similar energy to the tobacco industry strategies to increase sales of cigarettes in Indonesia. We have presented some new key strategies to bring about the elimination of smoking in Indonesia: ‘show as it is’, responsibility, economic incentives, career incentives, and advertising incentives. Hopefully, we can achieve at least one of two goals: helping people to give up smoking, or preventing them from starting to smoke.
